# Extremely premature infants born at 23–25 weeks gestation are at substantial risk for pulmonary hypertension

**DOI:** 10.1038/s41372-022-01374-w

**Published:** 2022-04-01

**Authors:** Hannes Sallmon, Martin Koestenberger, Alexander Avian, Friedrich Reiterer, Bernhard Schwaberger, Katharina Meinel, Gerhard Cvirn, Stefan Kurath-Koller, Andreas Gamillscheg, Georg Hansmann

**Affiliations:** 1 Department of Pediatric Cardiology, Charité—Universitätsmedizin Berlin, Berlin, Germany; 2 Department of Congenital Heart Disease/Pediatric Cardiology, Deutsches Herzzentrum Berlin (DHZB), Berlin, Germany; 3 European Pediatric Pulmonary Vascular Disease Network, Berlin, Germany; 4 Division of Pediatric Cardiology, Department of Pediatrics, Medical University Graz, Graz, Austria; 5 Institute for Medical Informatics, Statistics and Documentation, Medical University Graz, Graz, Austria; 6 Division of Neonatology, Department of Pediatrics and Adolescence Medicine, Medical University of Graz, Graz, Austria; 7 Physiological Chemistry Division, Otto Loewi Research Center, Medical University of Graz, Graz, Austria; 8 Department of Pediatric Cardiology and Critical Care, Hannover Medical School, Hannover, Germany

**Keywords:** Outcomes research, Diseases

## Abstract

**Objective:**

Extremely low gestational age newborns (ELGANs) represent an especially vulnerable population. Herein, we aimed to determine incidence and severity of pulmonary hypertension associated with bronchopulmonary dysplasia (BPD-PH) in extremely immature ELGANs (gestational age: 23^0/6^–25^6/7^ weeks).

**Methods:**

In this prospective observational cohort study, we assessed BPD-PH by means of several echocardiography markers and serum N-terminal pro-B-type natriuretic peptide (NT-proBNP) levels at 3 and 12 months of chronological age. In addition, we analyzed incidence and efficacy of pharmacologic treatment for BPD-PH.

**Results:**

At 3 months 15/34 ELGANs had echocardiographic evidence of BPD-PH, while at 12 months of age 6/34 still had PH. PH-targeted therapy consisted of sildenafil monotherapy in 11 and dual oral combination therapy (sildenafil and macitentan) in four ELGANs at 3 and 12 months.

**Conclusion:**

44% (15/34) of ELGANs developed BPD-PH. All received PH-targeted pharmacotherapy at 3 months, leading to hemodynamic improvements at 12 months in most infants.

## Introduction

With the improved survival of extremely low gestational age newborns (ELGANs), the long-term sequelae of prematurity, such as pulmonary hypertension (PH) associated with bronchopulmonary dysplasia (BPD-PH), are of emerging clinical interest [1, 2]. The pathogenesis of developmental lung disorders in BPD-PH is multifactorial (vascular, parenchymal, interstitial alterations) and incompletely understood. Prematurity, ventilatory support and other iatrogenic factors contribute to abnormal lung development including pulmonary vascular remodeling, ultimately leading to PH and right ventricular (RV) failure [3, 4]. Beside supportive care, an increasing number of patients with BPD receive PH-targeted pharmacotherapy (off-label in most instances) [5, 6]. About 25% of infants born <32 weeks gestation with moderate to severe BPD develop PH, and—10–20 years ago—almost half of these infants reportedly did not survive beyond the first 24 months of postnatal life [4, 7]. Study data specifically addressing BPD-PH in extremely premature infants (e.g., <26 weeks of gestation) are very sparse.

Here, we sought to investigate a cohort of ELGANs (23^0/6^–25^6/7^ weeks of gestational age) from birth to 12 months of chronological age. Echocardiographic variables of biventricular pressure, size and function were determined in preterm infants at risk for BPD-PH at 3 and 12 months of age. Since biomarkers may prove useful in guiding BPD-PH therapy, we determined serum N-terminal pro-B-type natriuretic peptide (NT-proBNP) levels as a secondary outcome measure in ELGANs up to 12 months of chronological age. We hypothesized that (i) chronic BPD-PH is common in ELGANs, (ii) can be delineated by a combinatory assessment of classical echocardiographic variables of RV pressure and function, and more recently published variables of RV/LV interaction and pulmonary blood flow, and (iii) improves with PH-targeted pharmacotherapy between 3 and 12 months of postnatal life.

## Methods

### Inclusion and exclusion criteria

We considered 47 consecutively born extremely immature infants (23^0/6^–25^6/7^ weeks gestational age) who were admitted to our NICU between January 2016 and December 2018 for inclusion. We excluded patients who died before 3 months of chronological age, surviving patients with congenital heart disease, structural lung abnormalities, genetic syndromes or other conditions expected to adversely affect life expectancy (*n* = 13). Resuscitation was not provided for infants <23 weeks’ gestational age. By 3 months postnatal age, we ultimately enrolled 34 surviving ELGANs into the study.

### Neonatal co-morbidities

Patients were treated according to institutional NICU protocols for invasive and noninvasive respiratory support. Postnatal steroids were administered intravenously for ≥7 days throughout the study period as a prophylaxis for BPD in infants who could not be weaned after 1 week of continuous mechanical ventilation (*n* = 18). For necrotizing enterocolitis (NEC) prophylaxis, a multimodal approach initiated within the first 24 h of life was followed, which included early trophic feeding with human breast milk, and enteral gentamicin, and *Lactobacillus casei rhamnosus*. Clinical and demographic parameters (e.g., gestational age, birthweight, length, and APGAR score) were recorded on electronic charts.

### Bronchopulmonary dysplasia (BPD)

BPD is classically defined as supplemental oxygen requirement at postnatal day 28 [8]. Severity of BPD was then categorized as mild (room air at 36 weeks of postmenstrual age, PMA), moderate (need for oxygen <30% at 36 weeks of PMA), or severe (need for oxygen ≥30% and/or positive pressure ventilation) [8]. We also included infants who did not fulfill these classic criteria to diagnose BPD (e.g., supplemental oxygen requirement on the 28th day of life), but required noninvasive respiratory support, thus fulfilling the more recent diagnostic requirements for BPD as outlined by Jensen et al. (*n* = 3) [9].

### Transthoracic echocardiography

Echocardiograms were performed on a standard echocardiographic system (Sonos iE33, Philips), by one experienced cardiologist, according to current guidelines [10–12]. Tricuspid regurgitation velocity (TRV)-derived right ventricular-to-right atrial systolic pressure gradient, a surrogate of RV systolic pressure (RVSP), and tricuspid annular plane systolic excursion (TAPSE) indicating RV longitudinal systolic function, were determined, as recently published in position papers [4, 13, 14]. A TRV > 2.5 m/s was considered the noninvasive cut-off value to define elevated pulmonary arterial pressure (PAP), in the absence of RV outflow tract obstruction [9, 10]. In order to accurately determine RVSP/sPAP from TRV (by using the simplified Bernoulli equation (dP = 4 · v^2^), the right atrial pressure (v-wave) would need to be added to the estimated pressure gradient (dP). However, since we did non perform invasive measurements of right atrial pressures, the provided RVSP values represent pressure gradient estimates derived from TRV interrogation added by an arbitrarily set atrial pressure value of 5 mmHG (dP = 4 · v^2^ + 5 mmHg). Severity of PH was categorized as mild, moderate or severe based on the estimated RVSP and stratified by the ratio of TRV-based RVSP and systolic systemic arterial pressure recorded simultaneously, (RVSP/SAP: <50% mild PH, 50–75% moderate PH, >75% severe PH). We evaluated variables of RV-LV interaction such as the RV/LV end-systolic diameter ratio and left ventricular end-systolic eccentricity index (LVesEI), and the pulmonary artery acceleration time (PAAT) as surrogate markers of PAP and PVR. Of note, in 6 patients RVSP could not reliably be estimated by TRV. However, these patients did not show any echocardiographic (TAPSE, PAAT, LVesEI, and RV/LV ratio) or clinical sign of RV dysfunction or increased PVR. Thus, these infants were included in the “no BPD-PH” group.

### Biomarker of cardiac wall stress

NT-proBNP was analyzed in venous blood samples at 3 and 12 months of chronological age (lithium heparin tubes) using the Cobas 8000 assay from Roche Diagnostics (Mannheim, Germany).

### Statistical analysis

Demographic variables are presented as absolute and relative counts, mean, and standard deviation (SD) or median and interquartile range (IQR), as appropriate. Comparisons of categorical characteristics between infants with and without BPD-PH were made using chi-square test or Fisher’s exact test, and for continuous variables using *t* test or Mann–Whitney *U* test, as appropriate. A *p* value of <0.05 was considered significant. Statistical analysis, including receiver operating characteristic (ROC) curve analysis, was performed using IBM SPSS Statistics 26.0.0 (SPSS Inc., IBM Company, Chicago, IL).

## Results

### Demographics and early mortality in ELGANs

**Fig. 1:**
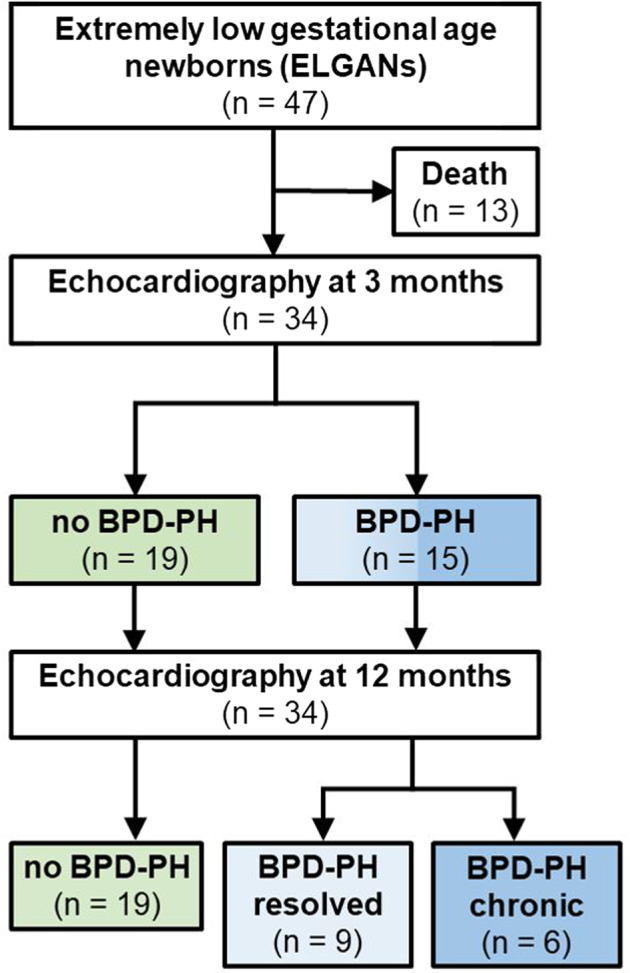
Study flow chart. BPD-PH pulmonary hypertension associated with bronchopulmonary dysplasia, ELGANs extremely low gestational age newborns.

Of the 47 eligible ELGANs born during the study period, 13 (27.7%, median gestational age 24.1 weeks) died within the first 3 months of postnatal life (median day of life 6, range 1–33), 7 of them (54%) on day of life 1 (Fig. 1). The following reasons were attributed to their deaths: severe lung hypoplasia (*n* = 3), sepsis with multiorgan failure (*n* = 4), perinatal asphyxia (*n* = 3), NEC (*n* = 1), cerebral malformation (lissenzephaly, *n* = 1), persistent seizures (*n* = 1), intraventricular hemorrhage, and intestinal perforation (*n* = 1). Fifteen of the remaining 34 patients (44%) showed echocardiographic signs of PH at 3 months, and six (17.6%) patients had PH signs at 12 months of chronological age. Detailed demographic and clinical patient characteristics are provided in Table 1.

**Table 1:** Demographics of ELGANs (23^0/6^–25^6/7^ weeks gestational age).

	All (*n* = 34)	BPD without PH (*n* = 19)	BPD-PH (*n* = 15)	*p* values
Sex (f/m)	16/18	10/9	6/9	0.537
GA (weeks)	24.6 (24.4–25.3)	24.9 (24.4–25.7)	24.6 (24.4–24.9)	0.215
BW (g)	680 (628–730)	726 (664–790)	650 (550–695)	0.004
SGA (*n*)	9	5	4	0.982
Apgar 5	8 (6–8)	8 (6–9)	7 (6–8)	0.758
Apgar 10	9 (8–9)	9 (8–9)	9 (7–9)	0.945
Maternal age (yrs)	29 (25–33)	28 (24–31)	31 (25–33)	0.421
Arterial cord blood pH	7.28 (7.22–7.34)	7.32 (7.26–7.38)	7.24 (7.18–7.31)	0.021
IRDS—I (grade)	3	3	0	0.436
IRDS—II (grade)	12	7	5	
IRDS—III (grade)	12	5	7	
IRDS—IV (grade)	7	4	3	
INSURE	14	10	4	0.127
LISA (*n*)	10	5	5	0.718
PDA ligature (*n*)	5	1	4	0.146
PPROM (*n*)	9	4	8	0.051
AIS (*n*)	8	2/19	6/15	0.100
EOS (*n*)	9	2/19	7/15	0.025
NCPAP (days)	46 (36–65)	37 (26–489)	65 (47–87)	<0.001
O_2_ (days)	58 (49–100)	50 (36–58)	103 (68–134)	<0.001
IMV (days)	15 (4–26)	7 (1–12)	29 (19–46)	<0.001
AirVo 2 at home	5	0	5	–
Hospital (months)	3.8 (3.2–4.5)	3.2 (2.8–3.7)	4.8 (3.8–5.1)	<0.001
Weight at discharge (g)	3362 (2952–3768)	3455 (3088–3836)	3270 (2790–3768)	0.271
PMA at discharge (weeks)	40.9 (39.6–43.6)	40.2 (39.0–40.7)	43.7 (42.7–45.1)	<0.001

Demographic data and perinatal risk factors.

*Airvo 2* nasal high-flow therapy, *AIS* amnion infection syndrome, *BPD-PH* pulmonary hypertension associated with bronchopulmonary dysplasia, *BW* Birth weight, *EOS* early onset sepsis, *GA* gestational age, *IMV* invasive mechanical ventilation, *INSURE* INtubation-SURfactant application-Extubation, *IRDS* infant respiratory distress syndrome, *LISA* less invasive surfactant application, *NCPAP* nasal continuous positive airway pressure, *O*_*2*_ oxygen, *PDA* persistent ductus arteriosus, *PMA* postmenstrual age, *PPROM* preterm prelabor rupture of membranes, *SGA* small for gestational age, *w/o* without, *yrs* years.

### Bronchopulmonary dysplasia (BPD)

31 of 34 ELGANs developed mild (*n* = 16), moderate (*n* = 10), or severe BPD (*n* = 5). By 3 months of age, PH was diagnosed in all infants with moderate (*n* = 10) or severe (*n* = 5) BPD (BPD-PH group), but not in patients with mild BPD (*n* = 16) or those without BPD (*n* = 3; no PH-group). Of note, the three infants who did not fulfill the classic criteria to diagnose BPD (e.g., supplemental oxygen requirement on the 28th day of life), all required noninvasive respiratory support by high-flow nasal cannula and/or CPAP, thus fulfilling the more recent requirements for diagnosing BPD (grade II) as outlined by Jensen et al. [9]. Therefore, we included these infants in the BPD group.

### Co-morbidities and incidence of PH

15/34 ELGANs (44%) showed signs of both PH and moderate-to-severe BPD by 3 months, and 6/34 (17.6%) infants still exhibited BPD-PH by 12 months of postnatal age. Of note, in six patients RVSP could be not reliably be estimated by TRV. However, these patients did not show any echocardiographic or clinical signs of RV dysfunction or increased PVR. Thus, these infants were included in the “no BPD-PH” group. TRV could reliably be interrogated in all infants with BPD-PH.

ELGANs with BPD-PH (*n* = 15) required supplemental oxygen for a longer time than those without PH (*n* = 19, 3 with mild BPD) [103 (68–134) days vs. 50 (36–58) days, *p* < 0.001]. Similarly, patients with BPD-PH received nasal continuous positive airway pressure (nCPAP) for a longer duration than those without PH [65 (47–87) days vs. 37 (26–48) days, *p* < 0.001], as was the case for the duration of invasive mechanical ventilation (iMV) [29 (19–46) vs. 7 (1–12); *p* < 0.05].

Of note, BPD-PH ELGANs showed a higher rate of early onset sepsis (EOS, 47%) as compared to patients without PH (11%, *p* < 0.05). In addition, ELGANs with BPD-PH exhibited a significantly lower birth weight than those with BPD in the absence of evidence of PH (mean BW: 650 g vs. 726 g; *p* < 0.004). However, there was no difference in the frequency of infants born small for gestational age (SGA) with PH (4/15) as compared to those without PH (5/19; *p* < 0.05). However, body weight at discharge was similar between patients with PH as compared to those without PH [3270 (2790–3768) vs. 3455 (3088–3836); *p* > 0.05]. Postmenstrual age at discharge was significantly higher in ELGANs with BPD-PH than in those without PH (43.7 vs. 40.2 weeks, *P* < 0.001). Consistently, BPD-PH patients required a longer hospital stay of 4.8 (3.8–5.1) months vs. 3.2 (2.8–3.7) months in patients without PH (*p* < 0.001), respectively.

### BPD severity is associated with PH and RV dysfunction

**Fig. 2:**
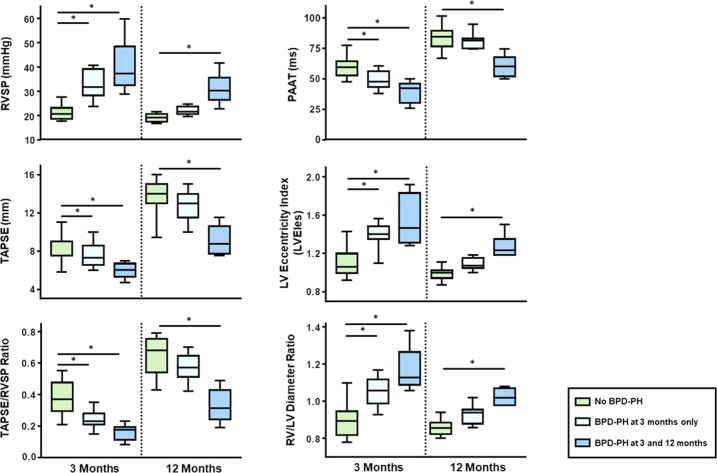
RVSP, TAPSE, RVSP/TAPSE, PAAT, LVesEI, and RV/LV ratio at 3 months and 12 months of chronological age in ELGANs without PH (*n* =  19, green boxes), ELGANs with BPD-PH at 3 months who showed resolution of PH at 12 months of age (*n*  =  9, light blue boxes), and patients who still had BPD-PH at 12 months of chronological age (*n*  =  6, blue boxes), *p value < 0.05. BPD-PH pulmonary hypertension associated with bronchopulmonary dysplasia, LVesEI left ventricular end-systolic eccentricity index, PAAT pulmonary arterial acceleration time, RV/LV ratio ratio of the RV basal diameter of the LV basal diameter, RVSP right ventricular systolic pressure, TAPSE tricuspid annular plane systolic excursion.

The 15 ELGANs with BPD-PH at 3 months of chronological age had a higher estimated “RVSP” of 34 (30–41) mmHg vs. 21 (19–23) mmHg in patients without PH (*p* < 0.001). At the 1-year follow-up time point, these 15 patents with BPD-PH diagnosed at 3 months still showed higher estimated RVSP values than infants without PH: 24 (22–28) mmHg vs. 19.5 (18–21) mmHg, respectively (*p* < 0.001). Compared to those without PH, the 15 ELGANs with BPD-PH had lower longitudinal systolic RV function [TAPSE 6.6 (6.0–8.0) mm vs. 9.0 (7.5–9.0) mm] at 3 months (*p* = 0.005)]. At 12 months of chronological age, and after PH-targeted pharmacotherapy, 9/15 ELGANs showed resolution of PH, while 6/15 infants continued to exhibit elevated RVSP values, indicating chronic PH. Of note, during 12 months follow-up, all 15 ELGANs who were initially diagnosed with BPD-PH had lower TAPSE values of 11.5 (9.0–13.5) mm vs. 14.0 (13.0–15.0) mm, compared to those without PH, respectively (*p* = 0.004). Accordingly, the TAPSE/RVSP ratio was lower in the BPD-PH vs. non BPD-PH group: 0.22 (0.17–0.23) vs. 0.37 (0.33–0.47) at 3 months (*p* < 0.001), and 0.50 (0.37–0.59) vs. 0.68 (0.57–0.75) at 12 months of chronological age (*p* = 0.008), respectively (Fig. 2, and Supplementary Table).

In addition, at 3 months, the 15 BPD-PH patients showed lower PAAT values of 45 (40–51) ms vs. 60 (53–65) ms in infants without PH, (*p* < 0.001). Again, lower PAAT values were also seen at 12 months of chronological age: 75 (66–82) ms vs. 85 (77–90) ms, (*p* = 0.01). The 15 BPD-PH patients had a higher LVesEI of 1.4 (1.32–1.50) vs. 1.06 (1.00–1.19) as compared to those infants without PH at 3 months of chronological age (*p* < 0.001). At 12 months of chronological age, all 15 ELGANs who showed signs of BPD-PH at 3 months of age still exhibited higher LVesEI values than patients without PH [1.17 (1.05–1.19) vs. 1.00 (0.94–1.02), respectively (*p* < 0.001)]. Furthermore, the 15 BPD-PH patients had an increased RV/LV end-systolic ratio of 1.09 (1.05–1.15) vs. 0.90 (0.82–0.93) than those infants without evidence of PH at 3 months of chronological age (*p* < 0.001), (Fig. 2, and Supplementary Table).

### Patients with persistent BPD-PH at 12 months of postnatal, chronological age

At 12 months of chronological age, 6/15 ELGANs still fulfilled the diagnostic criteria for BPD-PH. When compared to the no-PH group, these 6 ELGANs with chronic BPD-PH had higher RVSP [30.5 mmHg (28.0–34.0) vs. 21.0 mmHg (19.0–22.0), *p* < 0.001], higher LVEI [1.23 (1.18–1.30) vs. 1.03 (1.00–1.10), *p* < 0.001] and higher RV/LV ratio values [1.02 (0.98–1.07) vs. 0.87 (0.85–0.94), *p* < 0.001]. The 6 BPD-PH patients also had lower TAPSE [8.8 mm (7.8–10.3) vs. 13.8 mm (12.6–14.7), *p* < 0.001], lower PAAT [61 ms (53–66) vs. 83 ms (77–88), *p* < 0.001], and lower TAPSE/RVSP ratio values as compared to the infants without PH [0.32 (0.26–0.41) vs. 0.62 (0.52–0.72), *p* < 0.001] (Supplementary Table). Of note, all five infants who had severe BPD showed evidence of ongoing BPD-PH by 12 months of chronological age requiring treatment, while only one infant with moderate BPD had ongoing BPD-PH. These results indicate that infants with moderate BPD are more likely to show resolution of BPD-PH throughout the first 12 months of life.

### Medication

All ELGANs who were diagnosed with PH received PH-targeted pharmacotherapy. Side effects (e.g., flushing for sildenafil; or low red blood cell count, common cold-like symptoms for macitentan) of the drugs were not observed in our ELGAN cohort. At initiation of therapy no significant increase in heart rate or blood pressure drops of more than 10% to baseline were noted. At the first time point (3 months of chronological age) out of the 15 ELGAN with BPD-PH, 11 received sildenafil monotherapy and four patients with significant PH received a PH-targeted dual combination therapy with sildenafil and macitentan (start of dual combination therapy after a mean of 13.2 days under monotherapy). At the second time point (12 months of chronological age), out of the six ELGANs who still showed clear echocardiographic signs of BPD-PH, four infants received sildenafil monotherapy and two were still on dual oral combination therapy with sildenafil and macitentan, while in the other cases PH-targeted pharmacotherapy could be discontinued due to absent echocardiographic signs of PH and clinical improvement (Table 2).

**Table 2:** PH-targeted pharmacotherapy.

	**BPD-PH at 3 months (***n* = 15)	**Percentage of all ELGANs (***n* = 34)
Sildenafil monotherapy	11	32.4%
Sildenafil + macitentan combination therapy	4	11.8%
	**BPD-PH at 12 months** (*n* = 6)	**Percentage of all ELGANs** (*n* = 34)
Sildenafil monotherapy	4	11.8%
Sildenafil + macitentan combination therapy	2	5.9%

PH-targeted pharmacotherapy in ELGANs with BPD-PH.

*BPD-PH* pulmonary hypertension associated with bronchopulmonary dysplasia, *ELGANs* extremely low gestational age newborns.

### Biomarker of cardiac wall stress

**Fig. 3:**
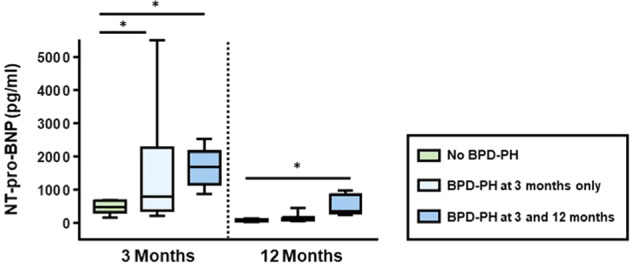
Changes in NT-proBNP at 3 months and 12 months of chronological age in ELGANs without PH (*n*  = 9, green boxes), ELGANs with BPD-PH at 3 months who showed resolution of PH at 12 months of age (*n*  = 9, light blue boxes), and patients who still had BPD-PH at 12 months of chronological age (*n*  =  6, blue boxes); *p value < 0.05. BPD-PH pulmonary hypertension associated with bronchopulmonary dysplasia, NT-proBNP N-terminal pro-B-type natriuretic peptide.

At 3 months of postnatal age, serum NT-proBNP concentrations were higher in infants with BPD-PH (median: 1255, IQR: 621–2135) as compared to those without PH (median: 466, IQR: 372–663 pg/ml; *p* = 0.023). Consistently, at the second time point (12 months) NT-proBNP values were still higher in the 6 ELGANs who had chronic BPD-PH despite pharmacotherapy (median: 359, IQR: 356–720 pg/ml vs. median: 87, IQR: 66–125 pg/ml; *p* = 0.001) (Fig. 3). A receiver operating characteristic (ROC) curve analysis revealed an area under the curve (AUC) of 0.91 (standard error 0.05) for NT-proBNP in discriminating patients with PH and those without BPD-PH, indicating an excellent diagnostic performance of NT-proBNP. Of note, none of the infants exhibited a significant intra- or extracardiac shunt (e.g., a hemodynamically significant patent ductus arteriosus), that might have confounded NT-proBNP results.

## Discussion

About 15–20 years ago, the incidence of PH in preterm infants born <32 weeks gestation had been reported to be ~25% [4, 15], with a PH-associated mortality of almost 50% at 24 months of chronological age [4, 7]. An even higher incidence of PH is suggested in ELGANs with respiratory morbidities such as BPD [16]. Based on systematic screening echocardiography at 3 months of uncorrected age, we diagnosed PH in patients with moderate-to-severe BPD but not in those with only mild BPD. We used traditional criteria for staging severity of BPD [8]. However, this definition has recently been challenged and a new definition of BPD was proposed [9]. The significance of this new, alternative BPD definition on the current incidence and morbidity/mortality of BPD-PH still needs to be investigated, but our results suggest that BPD as defined by Jensen et al. (e.g., no supplemental oxygen requirement, but noninvasive respiratory support) may not be associated with an increased risk of PH since in our cohort, BPD-PH was almost exclusively seen in infants with moderate-to severe BPD.

Recent pediatric PH guidelines recommend that all premature BPD infants should undergo an echocardiographic examination at 36 weeks gestational age and before hospital discharge (i.e., at 3 months of chronological age in ELGANs) for the evaluation of PH [4, 13, 17]. In addition to TRV and TAPSE as surrogate markers for RVSP and longitudinal systolic RV function, we herein investigated more recently published echocardiographic variables of ventricular-ventricular interaction such as RV/LV ratio and LVesEI, TAPSE/RVSP ratio (reflecting both functional capacity and the hemodynamic performance of the individual RV-PA unit) and PAAT (parameter used to estimate PA pressure). Recently, we established normative age-matched PAAT values in 756 healthy neonates and children and found reduced PAAT values in infants with significant PH [18]. In the current study, we found significantly shorter PAAT values in BPD-PH ELGANs as compared to ELGANs without PH at both time points (3 and 12 months of chronological age). Measurement of the end-systolic LVesEI (assessment of septal geometry) reflects ventricular-ventricular interaction [19]. It is unclear at which time point the preterm RV adapts in the context of moderate to severe lung disease (BPD) and subsequently increased pressure afterload. Usually, in patients with moderate-to-severe PH, the ratio of RV to LV pressure increases, and consecutively the septal curvature flattens. An association between LVesEI and BPD-PH has been reported, with LVesEI values of ≥1.0 indicating PH in premature infants [20]. In our study, BPD-PH ELGANs exhibited higher LVesEI values than those ELGANs without PH at both time points (3 and 12 months of chronological age).

Although therapeutic options for PH have increased over the past several decades, they remain limited and their use is usually not approved in preterm infants. Given the high mortality reported to be associated with BPD-PH, all ELGANs with features of BPD-PH in our cohort were treated off-label with PH-targeted medications, predominantly with the phosphodiesterase-5 inhibitor (PDE-5i) sildenafil and the endothelin receptor antagonist (ERA) macitentan. Among the ERAs, only bosentan has been approved for pediatric use in children older than 12 months (but not in ELGANs) by the European Medicines Agency (EMA) and the US Food and Drug Administration (FDA). Nevertheless, off-label use of sildenafil is increasing in premature infants with PH, but based on limited safety and efficacy data [21]. Compared to bosentan, macitentan treatment offers some potential benefits, such as a lower risk of drug interaction [22]. In addition, freedom from mandatory monthly liver function tests, as required under bosentan treatment, seems beneficial, especially in preterm neonates [23]. Experience with macitentan in the treatment of pediatric PH is sparse [24–26] and not available in ELGANs to date. We used sildenafil as first line treatment in all patients with signs of mild or moderate BPD-PH, and macitentan as an add-on drug only in those cases with signs of severe, resistant BPD-PH (e.g., >half-systemic PA pressure despite treatment). Our data may support the assumption that PH-targeted therapy is safe even in small ELGANs and can improve echocardiographic parameters of biventricular size and function at 12 months of chronological age, but require confirmation in larger systematic investigations.

Of our 34 ELGANs, 31 had BPD, but in fact, 19 of those 34 did not show clear signs of PH at 3 months of chronological age. Thus, even in ELGANs (23^0/7^–25^6/7^ weeks gestation), physiologically increased pulmonary vascular resistance values decrease to normal ranges in about half of the patients within the first 3 months of postnatal life [27]. However, the specific etiology of PH in at-risk infants remains unclear. Our results suggest that early inflammation (EOS) may play a role in PH etiology, while other specific risk factor still need to be investigated.

N-terminal pro-brain natriuretic peptide (NT-proBNP) levels were identified as significant prognostic marker in pediatric PH, deemed useful in the longitudinal assessment of PH patients [28]. Recent studies found a stable correlation between NT-proBNP and BPD development, with and without BPD-PH [29–31]. Accordingly, we found that NT-proBNP values at 3 and 12 months were higher in patients with BPD-PH as compared to those without PH, and well-suited for discriminating between infants with and those without BPD-PH. We therefore suggest that NT-proBNP provides a suitable addition to the diagnostic work-up for BPD-PH patients, as recently suggested in a novel treatment algorithm for BPD-PH [4].

### Limitations

While the sample size of this ELGAN cohort is limited, our results still allowed us to investigate standard and novel echocardiographic variables in extremely immature preterm infants (23^0/7^–25^6/7^ weeks’ GA) with and without BPD-PH. However, a comparative assessment and multi-regression analysis delineating the individual prognostic value of each single variable was not possible, since it is impossible to provide a “healthy” control group for a detailed statistical analysis. In addition, none of our hemodynamic results were confirmed by invasive measurements by cardiac catheterization, though this is not uncommon in such a vulnerable population, and actually an explicit exception in the recent EPPVDN recommendations on pediatric PH [4, 13]. In addition, this is a descriptive study that does not include any risk analyses of individual factors and their potential impact on BPD-PH development. Larger cohorts need to be studied to address these questions.

In conclusion, we show that ELGANs born at 23^0/7^–25^6/7^ weeks are at substantial risk for developing BPD-PH. At 3 months of age, 44% had signs of BPD-PH, while after 12 months of age, 18% still demonstrated echocardiographic features of BPD-PH requiring continuation of PH-targeted pharmacotherapy. While our findings may further implicate that early, sufficiently dosed PH-targeted pharmacotherapy (sildenafil ± ERA) can lower PA and RV pressure and has the potential to decrease mortality associated with BPD-PH, large controlled trials are required to assess safety and efficacy of PH-targeted therapy in the vulnerable population of preterm infants.

## Supplementary information

Supplemental Table

## Data Availability

The data that support the findings of this study are not publicly available due to their containing information could compromise the privacy of research participants but are available from the corresponding author (HS) upon reasonable request.
