# Supplementary material for: Extremely premature infants born at 23–25 weeks gestation are at substantial risk for pulmonary hypertension
Source: J Perinatol. 2022 Apr 1;42(6):781–7. doi: 10.1038/s41372-022-01374-w (PMC9184271; doi:10.1038/s41372-022-01374-w)
Supplement: Supplementary file 1 — Supplemental Table [file 41372_2022_1374_MOESM1_ESM.docx]

| **Supplemental Table: Echocardiographic data** | | | |
| --- | --- | --- | --- |
|  | **BPD without PH at 3 months** (n=19) | **BPD-PH at 3 months**  (n= 15) | **p-values** |
| **RVSP (mmHg)** | 21.0 (19.0 – 23.0) | 34.0 (30.0 – 41.0) | <0.001 |
| **TAPSE (mm)** | 9.0 (7.5 – 9.0) | 6.6 (6,0 – 8,0) | <0.001 |
| **PAAT (ms)** | 60 (53 – 65) | 45 (40 – 51) | <0.001 |
| **LVEI** | 1.06 (1.00 – 1.19) | 1.40 (1.32 – 1.50) | <0.001 |
| **RV/LV ratio** | 0.90 (0.82 – 0.93) | 1.09 (1.05 – 1.15) | <0.001 |
| **TAPSE/RVSP ratio** | 0.37 (0.33 – 0.47) | 0.22 (0.17 – 0.23) | <0.001 |
|  |  |  |  |
|  | **BPD without PH at 12 months** (n= 28) | **BPD-PH at 12 months**  (n=6) |  |
| **RVSP (mmHg)** | 21.0 (19.0 – 22.0) | 31.0 (28.0 – 34.0) | <0.001 |
| **TAPSE (mm)** | 13.8 (12.6 – 14.7) | 8.8 (7.8 – 10.3) | <0.001 |
| **PAAT (ms)** | 87 (85 – 94) | 61 (53 – 66) | <0.001 |
| **LVEI** | 1.03 (1.00 – 1.10) | 1.23 (1.18 – 1.30) | <0.001 |
| **RV/LV ratio** | 0.86 (0.83 – 0.88) | 0.96 (0.92 – 1.02) | <0.001 |
| **TAPSE/RVSP ratio** | 0.62 (0.52 – 0.72) | 0.32 (0.26 – 0.41) | <0.001 |

Legend: **Echocardiographic data of 34 ELGANs at 3 months and 12 months of chronological age**. *Abbreviations:* BPD-PH (=pulmonary hypertension associated with bronchopulmonary dysplasia); ELGANs (=extremely low gestational age newborns); LVEI (=left ventricular eccentricity index); PAAT (=pulmonary arterial acceleration time); RV (=right ventricle); RVSP (=RV systolic pressure); RV/LV ratio (=RV/left ventricular (LV) dimension ratio); TAPSE (=tricuspid annular plane systolic excursion); w/o (=without).
